# Integrated Plasma Metabolomics and Gut Microbiota Analysis: The Intervention Effect of Jiawei Xiaoyao San on Liver Depression and Spleen Deficiency Liver Cancer Rats

**DOI:** 10.3389/fphar.2022.906256

**Published:** 2022-07-18

**Authors:** Zhuoxian Li, Youxing Zhao, Jinlai Cheng, Lijing Xu, Xiaoyu Wen, Yuhao Sun, Meng Xia, Yining He

**Affiliations:** ^1^ School of Basic Medicine, Guangxi University of Chinese Medicine, Nanning, China; ^2^ Institute of Chinese Materia Medica, China Academy of Chinese Medical Sciences, Beijing, China

**Keywords:** liver cancer, metabolomics, gut microbiota, fecal microorganism, Jiawei Xiaoyao San

## Abstract

Primary liver cancer is the third most common malignancy, and hepatocellular carcinoma is its main subtype, with a high recurrence rate and high mortality. Intestinal microflora and metabolic disorders are present in most HCC patients. Traditional Chinese medicine (TCM) plays an important role in the composition of intestinal microorganisms and the transformation of active metabolites. Many scholars are trying to develop related drugs to assist in the treatment of liver cancer. In the preliminary study of the research group, it was found that the Jiawei Xiaoyao San has a certain therapeutic effect on liver cancer, but the specific mechanism is still unclear. Therefore, this study constructed a liver cancer rat model with liver stagnation and spleen deficiency, to explore the regulatory effect of Jiawei Xiaoyao San on plasma metabolites and intestinal microflora and to find the potential mechanism of Jiawei Xiaoyao San in the treatment of liver cancer. Plasma samples and fecal samples were collected from liver cancer rats with liver depression and spleen deficiency for microbiome 16S rDNA sequencing and metabolic ESI-QTRAP-MS/MS analysis. Various bioinformatics methods were used to analyze the dataset individually and in combination. The analysis and identification of plasma metabolomics showed that the intervention effect of Jiawei Xiaoyao San on liver cancer rats with liver depression and spleen deficiency was related to 11 differential metabolites and signal pathways such as primary bile acid biosynthesis, phenylalanine metabolism, pantothenate and COA biosynthesis, metabolic pathways, cholesterol metabolism, and bile secretion. Combined with fecal microbiological analysis, it was found that Jiawei Xiaoyao San could significantly change the composition of intestinal flora in liver cancer rates, increase beneficial bacteria, and reduce the composition of harmful bacteria. This study provides some experimental basis for the traditional Chinese medicine theory and clinical application of Jiawei Xiaoyao San in the adjuvant treatment of liver cancer. The potential mechanism may be to regulate metabolism and intestinal flora to play the role of regulating liver depression, activating blood, and detoxifying, to achieve the purpose of adjuvant treatment of liver cancer.

## GRAPHICAL ABSTRACT

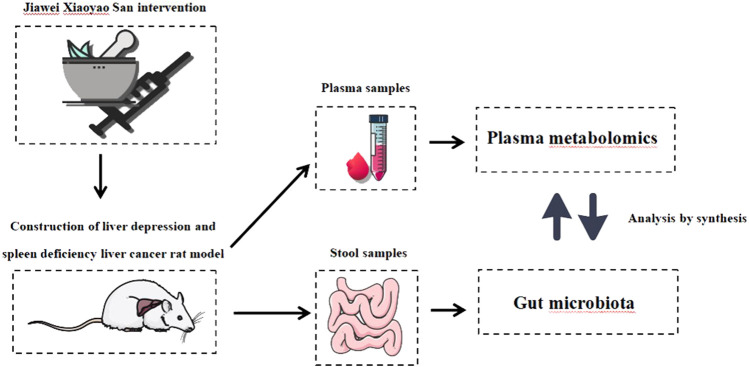

## Introduction

Liver cancer is a common and deadly malignant tumor that is the fourth leading cause of cancer-related death worldwide, with a poor prognosis and high mortality (Bi et al., 2021). According to the most recent World Health Organization data released in 2020 (Sung et al., 2021), the number of new cases of liver cancer in China has reached 413,300, ranking the first in the world, posing a serious threat to the lives and health of Chinese people. Although liver transplantation, surgical resection, radiofrequency ablation, and other treatments are available for early liver cancer, patients with liver cancer still face problems such as the low possibility of cure, high cost of treatment, poor prognosis, and low survival rate. It is still a key problem to seek effective, low toxicity, and economic treatment for liver cancer (Zhou et al., 2021).

At the same time, depression is one of the most common complications of liver cancer, accounting for 76.47 percent of cases (Cheng and Jing, 2018). Patients with liver cancer are more likely to experience anxiety, depression, and other negative emotions than healthy people . Also, depression will further promote the progression of liver cancer by promoting the proliferation and migration of liver cancer cells, which is closely related to the increased risk of death related to liver cancer (Xiang et al., 2018). In TCM, liver cancer could be classified into “hypochondriac pain” and “jaundice.” From the perspective of clinical syndromes, liver depression and spleen deficiency could be regarded as the most important syndromes. Depression belongs to the category of “depression syndrome” in ancient Chinese medical books; stagnation of liver qi is its important pathogenesis, and clinical treatment is mostly based on relieving liver depression. The participation of TCM in disease treatment is a unique diagnosis and treatment scheme in China, in which TCM compound is particularly good at the pharmacological effects of multi-components, multi-links, multi-levels, and multi-targets, which can play an overall regulatory role and has unique advantages.

Xiaoyao San was first nominated in The Song Dynasty Taiping Huimin Heji Fang, which is widely used in clinical treatment for treating liver stagnation, blood deficiency, and spleen weakness. At present, a large number of studies have confirmed that Xiaoyao San has definite efficacy in the treatment of depression, and its anti-depression mechanism is related to neurotransmitters and neurotrophic factors, hypothalamic–pituitary–adrenal axis, amino acids, lipids, inflammatory factors, and energy metabolism. The Jiawei Xiaoyao San used in this experiment is made from Xiaoyao San, which is derived from the empirical powder recorded by Yang (2012),, and the formula is as follows: 10 g *Angelica sinensis*, 10 g *Radix Bupleuri*, 10 g *Radix Codonopsis pilosulae*, 15 g *Radix Paeoniae Alba and Radix Paeoniae Rubra*, 10 g fried *rhizoma Atractylodis macrocephalae*, 15 g *Rhizoma Corydalis*, 6 g *Radix Glycyrrhizae*, 20 g *Radix Salviae Liguliobae*, 10 g *Cortex Moutan*, 10 g stir-fried two buds, 15 g *Poria cocos*, 15 g *Semen coicis*, 6 g *Fructus Gardeniae*, 15 g fried *Fructus Aurantii Immaturus*, and 15 g *Pericarpium Citri Reticulatae*.

The composition of “Xiaoyao San”: *Radix Glycyrrhizae*, *Angelica sinensis*, *Poria cocos*, *Radix Paeoniae Alba*, *rhizoma Atractylodis macrocephalae*, and *Radix Bupleuri. Radix Bupleuri* is used as the king medicine to soothe the liver and relieve depression and regulate liver qi; *Radix Paeoniae Alba* collects “Yin” in the soft liver; *Angelica sinensis* nourishes blood; *Poria cocos* and *Rhizoma Atractylodis macrocephalae* are used to strengthen the spleen and remove dampness; *Radix Glycyrrhizae* nourishes qi and slow liver, and the common use of the aforementioned drugs makes Xiaoyao San play the role in soothing the liver and relieving depression, nourishing blood and strengthening the spleen, and harmonizing the liver and spleen.

The “Jiawei Xiaoyao San” used in this experiment added some drugs based on “Xiaoyao San”: *Radix Codonopsis pilosulae* tonifies the spleen and qi; *Semen coicis* increases the strength of invigorating the spleen and oozes moisture; *Cortex Moutan* promotes blood circulation, removes blood stasis, clears heat, and cools blood; *Radix Paeoniae Rubra* disperses stasis, relieves pain, clears heat, and cools blood; *Radix Salviae liguliobae* invigorates blood and cools blood, removes stasis, and relieves pain; *Rhizoma Corydalis* promotes blood circulation, promotes qi, and relieves pain; fried *Fructus Aaurantii Immaturus* and *Pericarpium Citri Reticulatae* are combined to eliminate swelling and fill; *Fructus Gardeniae* clears heat and dampness, cools blood, and helps in detoxification; stir-fried *two buds* regulate qi-flowing for activating stagnancy. All of the drugs are used together to soothe the liver and strengthen the spleen, to activate qi and promote blood circulation, to resolve phlegm and detoxify, to promote dampness and cool blood, to eliminate distension, and to relieve pain. Jiawei Xiaoyao San often shows the intervention effect on depression, anxiety, and other emotional problems in the treatment of liver cancer (Yang, 2012; Wen and S, 2022).

Metabolomics is an emerging method in the field of systems biology, which has been widely used as a diagnostic tool in clinical and biological fields in recent years (Bujak et al., 2015). It can be used to analyze metabolites in biological tissues, cells, and fluids as a tool to explore potential biomarkers (Johnson et al., 2016). The balance and health of human body depend on the normal metabolic activities of intestinal microbiota (Visconti et al., 2019). The changes in intestinal microbiota can be caused by external environment such as diet and drugs and internal factors such as immune system and can also cause the corresponding changes in fecal metabolites (Visconti et al., 2019). In recent years, the combination of metabolome and microbiome studies has been recognized as one of the most prospective methods for assessing host–microbe interactions (Visconti et al., 2019).

At present, some studies have found that traditional Chinese medicine can play a role in the treatment of liver cancer and depression through metabolites and intestinal microorganisms (Lv and W, 2020; Li and F, 2022). However, at present, there are few studies on the treatment of liver depression and spleen deficiency liver cancer with Jiawei Xiaoyao San, and there are still no reports on its interaction with metabolome and fecal microorganisms.

In order to further investigate the intervention effect and possible mechanism of Jiawei Xiaoyao San on liver cancer with liver depression and spleen deficiency, a rat model of liver cancer with liver depression and spleen deficiency was established, along with metabolome and fecal microorganism analysis.

## Materials and Methods

### Materials

The medicinal supplies required for Jiawei Xiaoyao San were obtained from Guangxi University of Traditional Chinese Medicine‘s First Affiliated Hospital and used after being identified by the university’s traditional Chinese medicine identification room. SPF Sprague–Dawley male rats, weighing 200 ± 20 g, were purchased from Hunan Slake Jingda Experimental Animal Co., Ltd., Batch No. 4300470050925. Diethylnitrosamine with purity of 0.95 g/ml, Batch No: n84760-5ml, was purchased from Shanghai McLean Biochemical Technology Co., Ltd. On 13 February 2019, it passed the ethical review of animal experiments by the Ethics Committee of Guangxi University of Chinese Medicine, with the ethical approval number: DW20190213-016.

### Drug Preparation

Jiawei Xiaoyao San contains the following ingredients: 10 g *Angelica sinensis*, 10 g *Radix Bupleuri*, 10 g *Radix Codonopsis pilosulae*, 15 g *Radix Paeoniae Alba and Radix Paeoniae Rubra*, 10 g fried *rhizoma Atractylodis macrocephalae*, 15 g *Rhizoma Corydalis*, 6 g *Radix Glycyrrhizae*, 20 g *Radix Salviae Liguliobae*, 10 g *Cortex Moutan*, 10 g *stir-fried two buds*, 15 g *Poria cocos*, 15 g *Semen coicis*, 6 g *Fructus Gardeniae*, 15 g *fried Fructus Aurantii Immaturus*, and 15 g *Pericarpium Citri Reticulatae*. This experiment adopts the heating reflux method, which is an extraction method based on the extraction principles of comprehensive decoction method, wet percolation method, and reflux extraction method. The extraction and concentration can be carried out simultaneously. Compared with the decoction method, heating reflux has the advantages of accelerating the expansion of TCM decoction pieces, increasing the dissolution of soluble components and promoting the dissolution of active components (Zhong and W, 2021). The procedure is as follows: extraction with eight times of 85% ethanol is carried out, then heated and refluxed twice, filtered using a vacuum pump, combined the filtrate twice and fixed the volume for use, and concentrated the filtrate to a final volume of 3.55 g/ml (calculated according to the crude drug amount).

### Model Establishment

Sprague–Dawley rats were maintained under standard laboratory conditions (24 ± 1°C, 45 ± 15% relative humidity, and 12 h/12 h light/dark cycle), and food and water were freely available.

Liver cancer model (Zhong and W, 2021): After one week of adaptive feeding, 36 Sprague–Dawley rats were randomly divided into control group (CTL), model group (PLC), and Jiawei Xiaoyao San group (JWXY), with 12 rats in each group. The PLC group and JWXY group received intraperitoneal injection of 25 mg/kg diethylnitrosamine, three times a week for a total of 12 weeks, that is, 1–12 weeks.

Liver depression and spleen deficiency model (Yu, and Z, 2009): On the basis of a liver cancer model, chronic unpredictable mild stress (CUMS) stimulation was performed continuously for eight weeks at the fifth week after injecting diethylnitrosamine intraperitoneally. The CUMS method is performed by randomly taking one of the ten methods every day for eight weeks, including water prohibition for 24 h, fasting for 24 h, day and night reversal for 24 h, tilt 45° for 24 h, wet cushion for 24 h, horizontal shock for 45 min, tail clamping for 5 min, cold water swimming at 4°C for 5 min, environment at 45°C for 5 min, and behavior restraint for 4 h. Also, the same stimulus method is not allowed to appear continuously, and there is at least seven days of interval; the stimulation lasted for 8 weeks. That is, the liver depression and spleen deficiency model was established synchronously at 5–12 weeks on the basis of liver cancer modeling.

**FIGURE 1:**
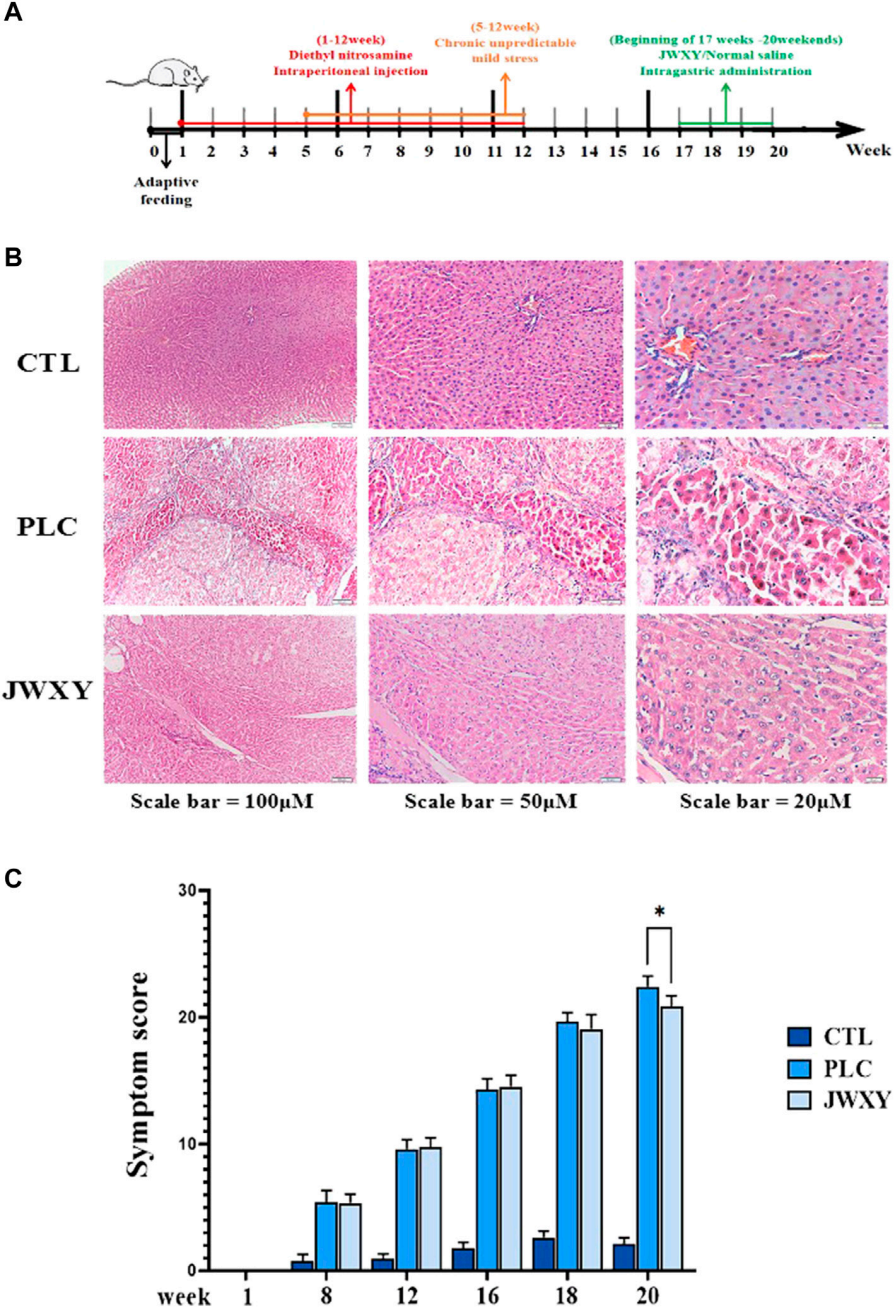
**(A)**: Process diagram of liver cancer model of liver depression and spleen deficiency. **(B)** Liver HE staining of CTL, PLC, and JWXY groups at different magnifications (bar = 100/50/20 μM). **(C)** Symptom score of the three groups was 0 in the first week, and the symptom score of PLC and JWXY groups was significantly higher than that of the CTL group in the eighth week. The symptom score of the JWXY group was lower than that of the PLC group in the 18th week and was significantly lower than that of the PLC group in the 20th week (*p* < 0.05).

On the first day of the 17th week, the JWXY group was treated with 6 ml/kg of Jiawei Xiaoyao San by gavage, once a day, for four consecutive weeks. The other groups were given the same amount of normal saline by gavage. Samples were taken on the last day of week 20 (Figure 1A).

### Symptom Score

The rat model of liver depression and spleen deficiency + liver cancer constructed this time is a disease combination model with both disease and symptom characteristics under the guidance of the traditional Chinese medicine theory. Among them, liver depression and spleen deficiency syndrome is a common type of syndrome in TCM clinical practice, and it mainly refers to liver dysfunction and dysfunction of the spleen in transport, showing the main symptoms of chest and flank pain, abdominal distension, and loose stools, etc. With the liver controlling dispersion, liver-qi depression could not be delivered normally, and also spleen-qi could not be operated normally. As a result, weakened digestive function, lack of appetite and numb to react, abdominal fullness and distention, and limb burnout, etc., appear (Zhao and L., 2018).

The clinical judgment of liver depression and spleen deficiency syndrome is based on the dialectical analysis of traditional Chinese medicine doctors on the basis of seeing, hearing, asking, and making four diagnoses. At present, the clinical judgment of the syndrome of liver depression and spleen deficiency mainly refers to the corresponding TCM syndrome scale, which summarizes the typical manifestations of the syndrome of liver depression and spleen deficiency. The symptoms and signs were scored quantitatively, and then the severity of the syndrome type was judged according to the occurrence rate and score of the indication. The higher the score, the more serious the symptom will be.

This semi-quantitative symptom score was also used in this study. According to the syndrome score of liver depression and spleen deficiency in guiding principles of TCM clinical research (Zheng, 2002), combined with the animal syndrome score table of liver depression and spleen deficiency, the symptoms are provided with a score (Sun S, 2017). By observing the symptoms and signs of rats in each group every week and scoring the symptoms, the severity of the syndrome of liver depression and spleen deficiency in each group was evaluated.

**Table:** 

List 1 Quantitative scale of rat syndrome grading				
Symptom	Asymptomatic 0	Light 1	Medium 2	Heavy 3
Abdominal swelling	No change	Mild swelling	Moderate swelling	Severe swelling
Food intake	No change	Mild reduction	Moderate reduction	Severe reduction
Water intake	No change	Mild reduction	Moderate reduction	Severe reduction
Depression or mania	No change	Mild abnormal	Moderate abnormal	Severe abnormal
The hair dry	No change	Mild boring	Medium boring	Severe boring
Loose stool	No change	Mild loose stools	Medium loose stools	Severe loose stools
Eye color	Red gold	Light red	Deep red	Dark red
Tongue nature	Light red	Dark red	Dark red slant purple	Cyan purple with ecchymosis

### The Sample Collection

After the experiment, rats were anesthetized with 3% pentobarbital sodium (0.01 ml/kg) through intraperitoneal injection, and the blood samples were collected from the abdominal aorta and centrifuged after 4 h of rest. The upper portion of serum was collected and stored at −80°C for standby. After collecting the blood, the rats in each group were killed, and their liver tissues were taken, fixed with 3% paraformaldehyde, and stored at −80°C. The feces of each group were collected and frozen at −80°C after liquid nitrogen quick freezing treatment.

### Hematoxylin-Eosin Staining

The liver tissues fixed in 3% paraformaldehyde were removed, paraffin-embedded, sectionalized, and hematoxylin-eosin staining (HE) was performed to observe the pathological changes of liver tissues in each group.

### Plasma Metabolomics

To ensure that blood samples were evenly mixed, they were retrieved from the −80°C refrigerator and thawed on ice for 10 seconds. Pre-chilled methanol, containing internal standard 2-chlorophenylalanine, was added, vortically swirled at 1,200 r/min for 3 min, and centrifuged at 4°C for 10 min. After centrifugation, the supernatant was taken into the EP tube and centrifuged at 4°C for 5 min at 12,000 r/min.

The supernatant was taken into the inner tube of the injection bottle for subsequent analysis. The data acquisition instrument system mainly uses ultra-high-performance liquid chromatography (UPLC) and tandem mass spectrometry (MS/MS). The liquid phase consisted of Waters ACQUITY UPLC HSS T3 C18 (1.8 µm, 2.1 mm*100 mm); mobile phase: A-phase ultrapure water (0.04% acetic acid) and B-phase acetonitrile (0.04% acetic acid); the elution gradient was 0 min water/acetonitrile (95:5 V/V), 11.0 min 5∶95 V/V, 12.0 min 5∶95 V/V, 12.1 min 95∶5 V/V, and 14.0 min 95∶5 V/V. The flow rate was 0.4 ml/min, the column temperature was 40°C, and the injection volume was 2 μl. The mass spectrometry conditions mainly include a 500°C electrospray ion source (ESI); 5,500 V mass spectrum voltage, -4500V; ion source gas I (GSI) 55 psi, gas II(GSII) 60 psi, and curtain gas (CUR), 25psi; and collision-induced ionization (CAD) parameter is set to high. In the triple quadruple (Q-Trap), each ion pair is scanned and detected according to the optimized cluster removal voltage (DP) and collision energy (CE) (Chen et al., 2013). The data analysis is based on the MWDB (MetWare database) established by Wuhan Metware Biotechnology Co., Ltd. The information and secondary spectrum data are qualitatively analyzed by detecting substance retention time (RT) and parent and daughter ions. The metabolic quantification was completed by triple quadruple mass spectrometry (Fraga et al., 2010), and the mass spectrometry data were processed by Analyst1.6.3 and Multi Quant software. Multivariate statistical analysis was used to establish a digital model to summarize the characteristics of the metabolic spectrum. Principal component analysis (PCA) (Chen et al., 2009) was used to compare the total metabolic differences and intra-group variability among different groups. R (www.r-project.org/) was used to cluster analysis of the accumulation patterns of metabolites among different samples, and the content data of metabolites were normalized based on the range method. The total difference of metabolites between groups was analyzed by orthogonal partial least squares discrimination analysis (OPLS-DA). Multivariate analysis of OPLS-DA model variable importance projection (VIP) was used to screen differential metabolites. The VIP value and fold change in the OPLS-DA model were analyzed by univariate analysis to further screen out the metabolites with significant differences of VIP≥1, fold change ≥2, or fold change ≤0.5. The KEGG database was used for functional annotation and pathway enrichment of differential metabolites.

### Gut Microbiota

According to the manufacturer’s instructions, DNA was extracted from stool samples using the E. Z.N.A.®Stool DNA Kit, and the extracted quality of DNA was tested by agarose gel electrophoresis and was determined using a UV spectrophotometer. Fecal flora was sequenced using 16S rDNA, and the variable region of the rDNA gene was amplified by primers designed according to the conserved region of ribosomal RNA to analyze microbial diversity. The amplified primers were universal primers for the V4 region of bacterial 16S rDNA (Fadrosh et al., 2014): 515F GTGYCAGCMGCCGCGGTAA and 805R GACTACHVGGGTATCTAATCC. The universal connector and sample-specific barcode sequence were added to the universal primers for PCR amplification of the rDNA gene variable region (V4).The amplified products were recovered using 2% agarose gel electrophoresis, and AMPure XT Beads were recycled. The purified PCR products were quantified using Qubit, and the library was prepared and checked. MiSeq sequencer was used for 2 × 300 bp double-terminal sequencing. The results obtained by sequencing were preprocessed according to barcode information, such as data splitting, double-end splicing, and chimera filtering, to obtain clean data. Results of OTU clustering were used to ensure the accuracy of analysis, and OTU abundance of different samples was obtained to evaluate microbial diversity, richness, and community stability. Beta diversity analysis was conducted based on OTU abundance to compare the similarities and differences in species composition between groups. The OTU representative sequence was compared with the 16S rDNA database. The species abundance tables of different kingdoms, phyla, classes, orders, families, genera, and species were obtained to carry out composition analysis, test different species between groups, and distinguish the important flora (biomarker) affecting different groups.

## Results

### Histopathological Morphology of the Liver and Changes of Symptoms and Signs

After being induced by diethylnitrosamine combined with chronic mild and unpredictable stress, HE staining results showed that the liver tissue of the CTL group had complete cell structure, uniform size, clear nucleus, and hepatic cord radially arranged around the central vein. HE staining in the PLC group showed that the hepatic lobule structure was damaged, and there were dysplastic cells, binuclear cells, and inflammatory cells in the hepatic lobule area. The pathological changes of liver tissues in hepatocellular carcinoma rats with liver depression and spleen deficiency in the JWXY group were alleviated, the hyperplastic cells became atypically smaller, binuclear cells decreased, and inflammation was alleviated (Figure 1B). Jiawei Xiaoyao San can significantly improve the symptoms and quality of life of liver cancer rats (Figure 1C). Preliminary *in vivo* results of this study have been published (Cheng and W, 2020). Based on the aforementioned *in vivo* experiments, this study further explored the intervention effect and related mechanism of Jiawei Xiaoyao San on liver depression and spleen deficiency liver cancer rats.

### Results of Principal Component Analysis and Orthogonal Partial Least Squares-Discriminant Analysis

**FIGURE 2:**
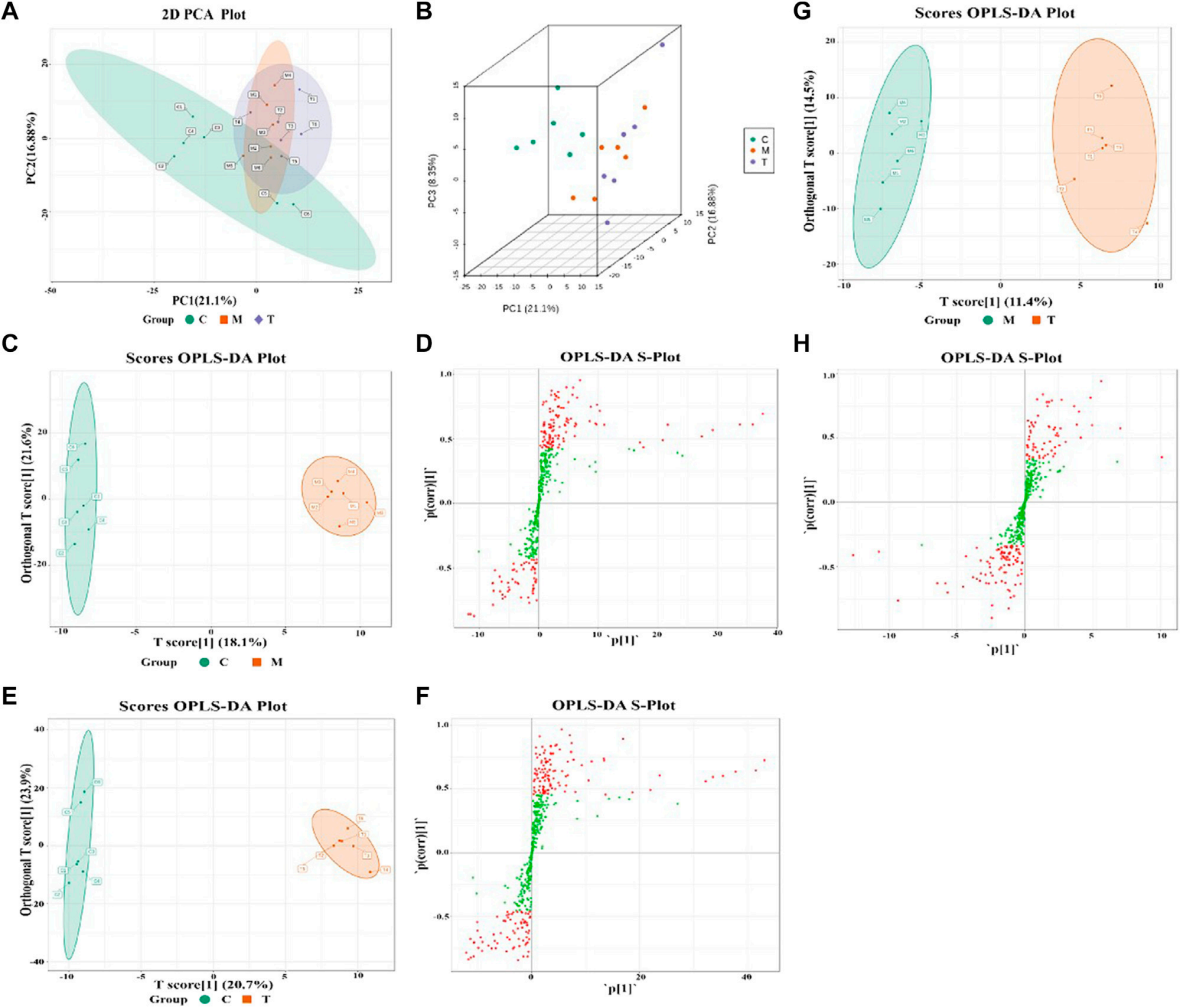
C is the CTL group; M is the PLC group; and T is the JWXY group. **(A–B)** Principal component analysis (PCA) results of CTL, PLC, and JWXY groups; **(C)** OPLS-DA score map of the CTL group and PLC group; **(D)** OPLS-DA S-plot of the CTL group and PLC group; the abscissa represents the correlation coefficient of the main component and metabolite, and the correlation coefficient is represented by the ordinate. The metabolites in the upper right and lower left corners showed the most significant differences. The red dot indicates the metabolite VIP value >1, while the green one indicates the metabolite VIP value <1; **(E–F)** OPLS-DA score plot and OPLS-DA S-plot of the CTL group and JWXY group; **(G–H)** OPLS-DA score and OPLS-DA S-plot of the PLC group and JWXY group.

Principal component analysis was performed on the samples requiring difference analysis in each group to observe the degree of variability between groups. In addition, using the partial least squares-discriminant analysis (PLS-DA) maximization region grouping, combining orthogonal signal correction and the PLS-DA method, the X-matrix information was decomposed into two types of y-related and y-unrelated information, and irrelevant differences and filter difference variables were removed. Figures 2A–B show the two-dimensional and three-dimensional spatial distribution of principal components in each metabolic spectrum. PCA analysis results showed that the metabolic spectrum distribution of different groups had a discrete trend, with certain differences.

### Screening Results and Analysis of Differential Metabolites

**FIGURE 3:**
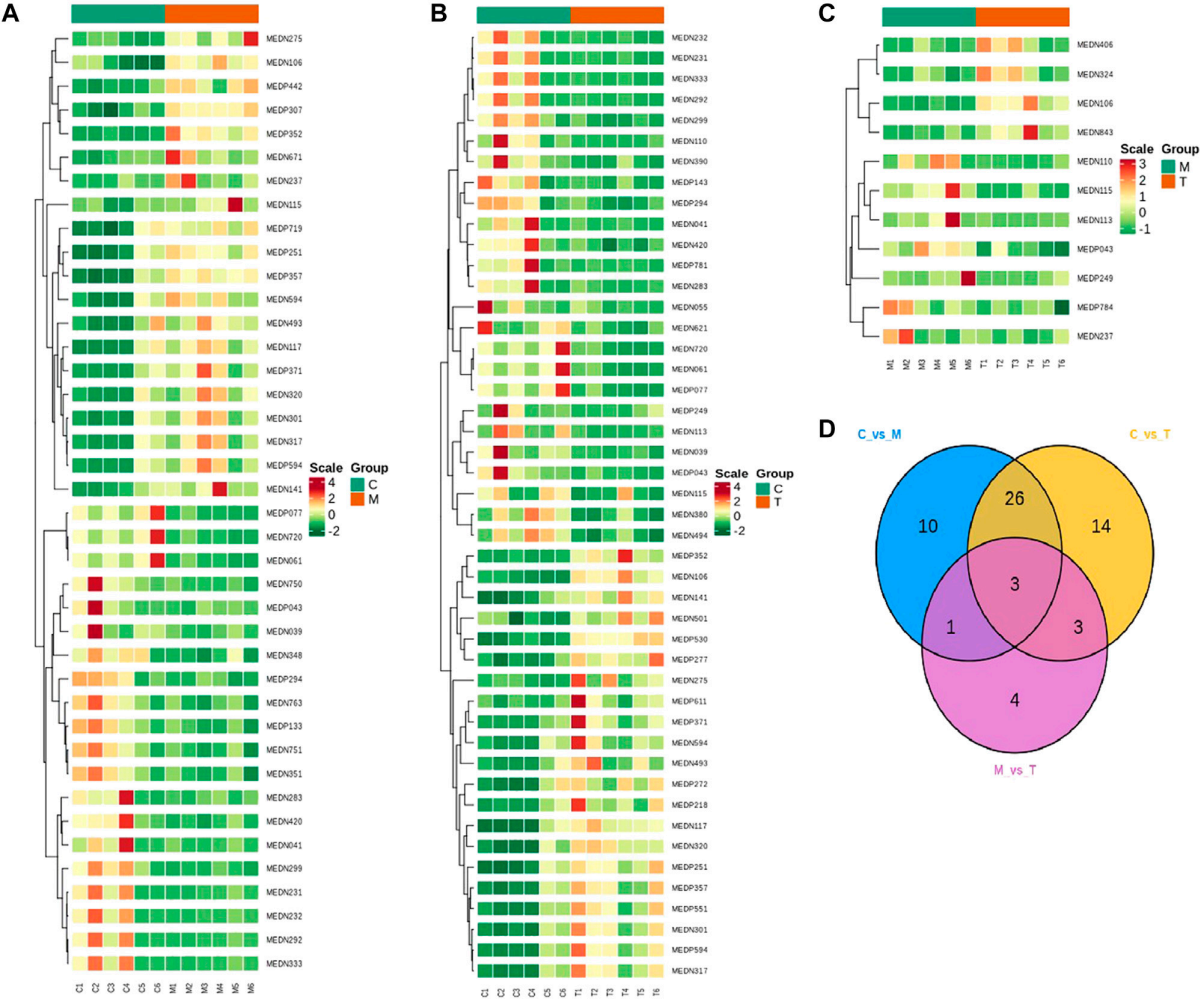
C is the CTL group; M is the PLC group; and T is the JWXY group. **(A)** Clustering heat map of metabolites with significant difference between the CTL group and PLC group. **(B)** Clustering heat map of metabolites with significant difference between the JWXY group and CTL group. **(C)** Clustering heat map of metabolites with significant difference between the JWXY group and PLC group. Red indicates a strong positive correlation, and green indicates a strong negative correlation. **(D)** Venn diagram of differential metabolites between groups.

Based on the results of PCA and OPLS-DA, the metabolites with fold change ≥2 or fold change ≤0.5 and VIP ≥1 were screened as differential metabolites, and 97 differential metabolites were detected. Compared with the CTL group, the PLC group had 40 differential metabolites such as glutathione reduced form, of which 20 were downregulated and 20 were upregulated, mainly involving organic acids, amino acids, carbohydrates, oxidized lipids, bile acids, phenols, benzene, and other metabolic categories. Compared with the CTL group, glycochenodeoxycholic acid and other 46 metabolites were found in the JWXY group, including 25 downregulated and 21 upregulated metabolites, mainly involving organic acids, amino acids, bile acids, carbohydrates, lipids and other fatty acids, coenzymes, vitamins, and benzene. Compared with the PLC group, the JWXY group had 11 differential metabolites such as chenodeoxycholic acid, among which seven were downregulated and four were upregulated, mainly involving bile acids, amino acids, organic acids, carbohydrates, coenzymes, vitamins, aldehydes, and other metabolic categories (Table 1; Figure 3). The metabolites with significant differences were normalized, and the change rule of metabolites was observed by drawing a cluster heat map and Venn diagram (Figure 3).

**TABLE 1:** Statistical table of differential metabolites.

Group	Total sig metabolites	Downregulated	Upregulated
CTL vs. PLC	40	20	20
CTL vs. JWXY	46	25	21
PLC vs. JWXY	11	7	4

**Note:** Group: grouping information; total sigmetabolites: the number of metabolites with a significant difference; downregulated: the number of downregulated metabolites; upregulated: the number of upregulated metabolites.

### Results of Metabolic Pathway Analysis

**FIGURE 4:**
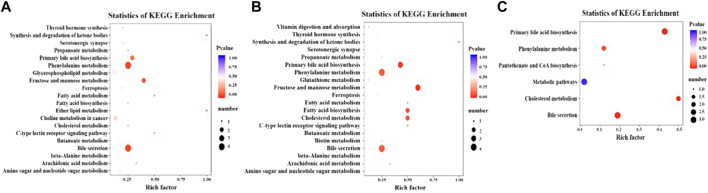
**(A)** KEGG enrichment diagram of CTL and PLC comparison groups; **(B)** KEGG enrichment of CTL and JWXY groups; **(C)** KEGG enrichment diagram of PLC and JWXY comparison groups. The abscissa represents the rich factor corresponding to each pathway, the ordinate represents the pathway name, and the color of the point is the *p*-value. The redder the point is, the more significant the enrichment is. The size of the dot represents the number of enriched differential metabolites.

The KEGG pathway is enriched based on the results of differential metabolites. The rich factor is the ratio of the number of differentially expressed metabolites in the corresponding pathway to the total number of metabolites detected and annotated in the pathway, and the ratio is positively correlated with the degree of enrichment. The enrichment results showed that, when the CTL group was compared with the PLC group, the differential metabolic pathways mainly involve primary bile acid biosynthesis, phenylalanine metabolism, fructose and mannose metabolism, choline metabolism in cancer, cholesterol metabolism, and bile secretion, etc. When the CTL group was compared with the JWXY group, the differential metabolic pathways mainly involve primary bile acid biosynthesis, phenylalanine metabolism, fructose and mannose metabolism, fatty acid biosynthesis, cholesterol metabolism, and bile secretion. When the PLC group was compared with the JWXY group, the differential metabolic pathways mainly involve primary bile acid biosynthesis, phenylalanine metabolism, metabolic pathways, cholesterol metabolism, and bile pituitary, etc. (Figure 4).

### Analysis of Intestinal Microbial Composition and Diversity

**FIGURE 5:**
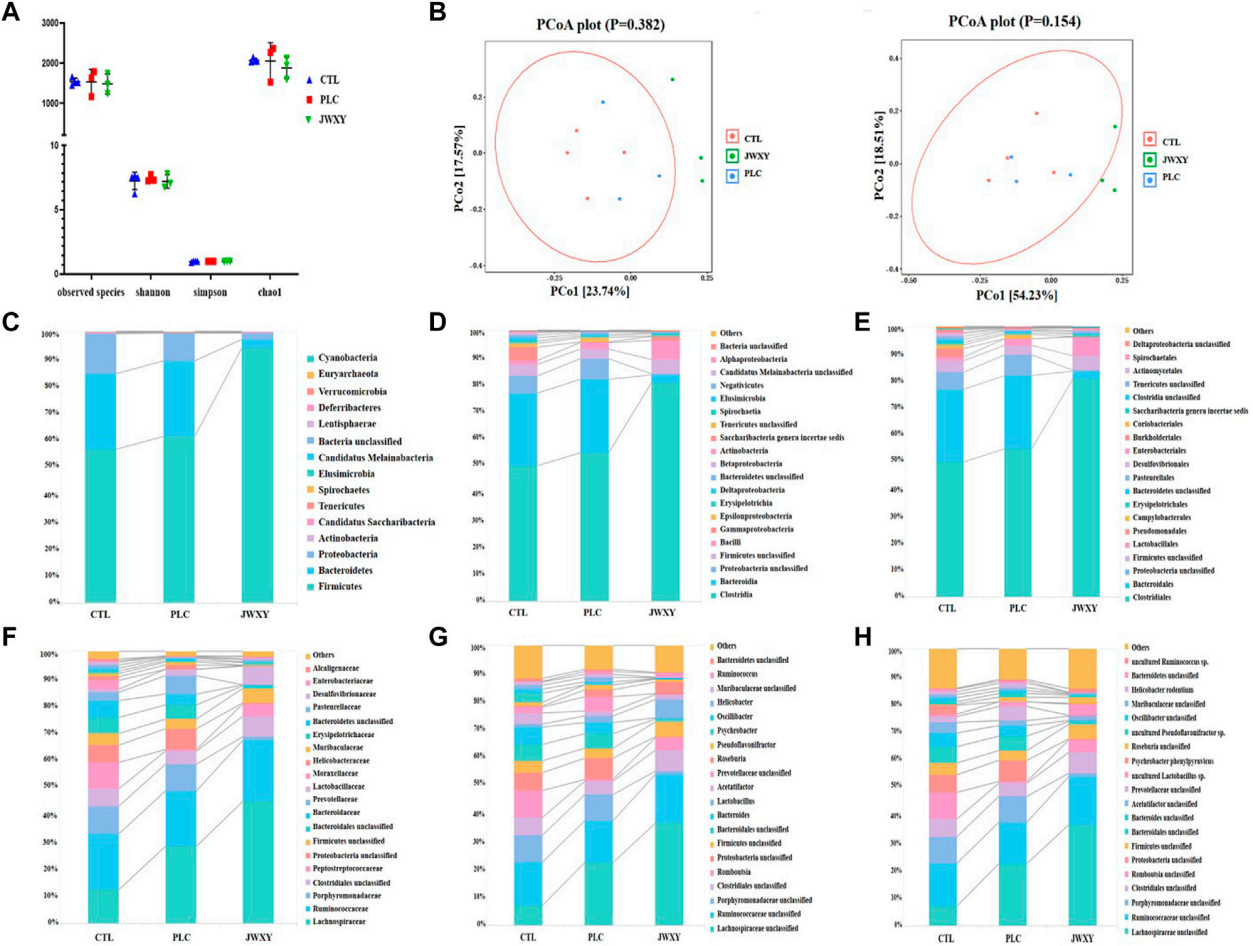
Proportion of intestinal microbe in CTL, PLC, and JWXY groups. **(A)** Alpha diversity index among the three groups. **(B)** Beta diversity distance PCoA analysis (unweighted and weighted). **(C)** Composition proportion of bacteria phylum in each group. **(D)** Composition proportion of bacteria class in each group. **(E)** Composition proportion of bacteria order in each group. **(F)** Composition proportion of bacteria family in each group. **(G)** Composition proportion of bacteria genus in each group. **(H)** Composition proportion of bacteria species in each group.

The differences in intestinal microbial diversity between groups were evaluated by alpha and beta diversity. Alpha diversity includes observed species, Shannon index, Simpson index, and Chao1; species richness information is reflected by the observed species and Chao1 index, while species richness and evenness are reflected by Shannon and Simpson indexes. There was no significant difference between groups (Figure 5A). The PCoA diagram of unweighted and weighted beta diversity distance between the three groups showed a discrete trend among the three groups (Figure 5B). As shown in Figures 5C–H, each group showed different microbial assemblages at phylum, class, order, family, genus, and species levels (the top 20 classifications with the highest abundance were selected). At the phylum level, Firmicutes, Bacteroidetes, and Proteobacteria were the main microbial groups. At the family level, the three microbial assemblages were mainly Lachnospiraceae, Ruminococcaceae, and Porphyromonadaceae. At the genus level, the three groups of microbial assemblages were mainly Lachnospiraceae unclassified, Ruminococcaceae unclassified, and Porphyromonadaceae unclassified. The composition of intestinal microorganisms in phylum, family, and genus of each group is shown in Table 2.

**TABLE 2:** The Composition of intestinal microorganisms in phylum, family, and genus of each group.

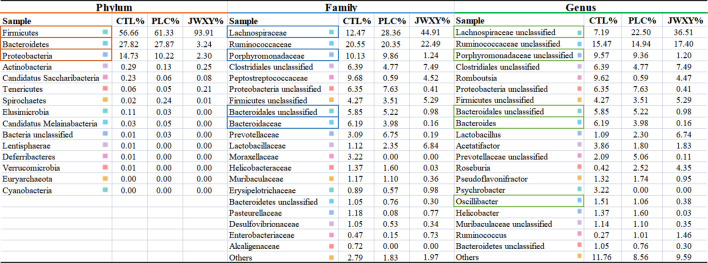

**Note:** The orange boxes represent the microorganisms with significant changes in the phylum, the blue boxes represent the microorganisms with significant changes in the family, and the green boxes represent the microorganisms with significant changes in the genus (*p* < 0.05).

In the phylum level of intestinal microorganisms in each group, the three groups of microbial combinations were mainly Firmicutes, Bacteroidetes, and Proteobacteria. After the intervention of the Jiawei Xiaoyao San, the expression level of Firmicutes was greatly increased, while the expression level of Bacteroidetes was significantly decreased. Compared with the PLC group, the Proteobacteria level in the JWXY group decreased significantly. *p* < 0.05 was considered a significant difference. In the level of intestinal microbial families in each group, Lachnospiraceae in the PLC group and JWXY group was significantly increased with a significant difference, while the content of Lachnospiraceae in the JWXY group also showed an increasing trend when compared with the PLC group, but there was no significant difference (*p* > 0.05). The contents of Porphyromonadaceae in the PLC group and CTL group were higher than those in the JWXY group, and the difference was significant. In the comparison of Bacteroidales unclassified content, the content of Bacteroidales unclassified in the PLC group and CTL group was higher than that in the JWXY group, with a significant difference. Bacteroidaceae in the JWXY group was significantly lower than that in the PLC group. In the genus level of intestinal microbes in each group, the CTL group was compared with the PLC group, and Lachnospiraceae unclassified in the PLC group increased significantly. Compared with the PLC group, Porphyromonadaceae unclassified, Bacteroides, and Oscillibacter in the JWXY group decreased significantly, with a significant difference. The JWXY group was compared with the CTL group. In the JWXY group, the contents of Lachnospiraceae unclassified, Porphyromonadaceae unclassified, Bacteroidales Unclassified, Bacteroides, and Oscillibacter were decreased.

## Discussion

The “liver depression and spleen deficiency liver cancer rat model” established in this experiment is a disease combination model with disease and symptom characteristics under the guidance of traditional Chinese medicine theory. In the theory of traditional Chinese medicine, the generation of liver depression depends on emotional paralysis, liver qi stagnation, sudden emotional stimulation or the invasion of a foreign evil spirit and bring about the liver to lose catharsis, and reach the Qi mechanism of the body. Based on the relationship between the liver and spleen in the Five Elements Theory, the stagnation of liver wood can invade spleen soil, which leads to the pathological evolution of liver depression and spleen deficiency. Some researchers have successfully copied and verified the establishment of the model of liver depression and spleen deficiency by flexibly using the theory of traditional Chinese medicine (Yue and D, 2008). In addition, the modeling method of the model liver depression and spleen deficiency has been confirmed in other studies (Luo et al., 2007; Liang and Guo, 2010). Therefore, the establishment of the model of liver depression and spleen deficiency referred to this modeling method. Moreover, liver depression and spleen deficiency belong to the category of TCM syndrome diagnosis, which is mainly judged by the four diagnoses of TCM, which have certain subjectivity and limitations. At present, the syndrome score scale is mostly used to judge the corresponding symptoms (Zhao and W, 2008). All of the symptoms listed on the scale are typical of the syndrome of liver depression and spleen deficiency. The symptoms and signs were quantitatively scored, and the severity of the syndrome type was determined based on the occurrence rate and score of the indication. The more severe the symptom is, the higher the score is. The severity of the syndrome of liver depression and spleen deficiency in each group was evaluated by observing and scoring the symptoms of rats in each group every week. The intervention of Jiawei Xiaoyao San can reverse the plasma metabolites of L-3-phenylacetic acid, 3-(3-hydroxyphenyl)propionate acid, N-amino-L-aspartate, taurochenodeoxycholic acid, D-glucuronic acid, glutathione oxidized, glycoursodeoxycholic acid, glycochenodeoxycholic acid, and chenodeoxycholic acid. The composition of intestinal microbes in liver depression and spleen deficiency liver cancer rats is adjusted, and the contents of Proteobacteria, Porphyromonadaceae, Bacteroidales unclassified, Bacteroidaceae, Porphyromonadaceae_unclassified, Bacteroides, and Oscillibacter are reversed.

The metabolomics results showed that when compared with the PLC group, the differential metabolites in the JWXY group intervened by Jiawei Xiaoyao San showed an upregulated trend, including L-3-phenylacetic acid, 3-(3-hydroxyphenyl)propionate acid, N-amino-L-aspartate, and taurochenodeoxycholic acid; also, differential metabolites showed a downregulated trend such as D-gluconic acid, glutathione oxidized, glycoursodeoxylic acid, glycochenodeoxycholic acid, and chenodeoxycholic acid. Compared with the CTL group, the JWXY group intervened by Jiawei Xiaoyao San showed an upward trend of differential metabolites, including 5-hydroxyindole-3-acetic acid, methyl indole-3-acetate, N-acetyl-L-histidine, D-mannitol, and acetyl tryptophan. It is worth noting that D-glucuronic acid and chenodeoxycholic acid were upregulated in the differential metabolites of the PLC group and CTL group but were downregulated in the PLC group and JWXY group. The analysis of differential metabolic pathways showed that the differential metabolic pathways between CTL and PLC groups were mainly involved in primary bile acid biosynthesis, phenylalanine metabolism, fructose and mannose metabolism, choline metabolism in cancer, cholesterol metabolism, and bile secretion, etc. CTL and JWXY groups’ metabolic pathways were mainly involved in primary bile acid biosynthesis, phenylalanine metabolism, fructose and mannose metabolism, fatty acid biosynthesis, cholesterol metabolism, and bile secretion. PLC and JWXY groups were mainly involved in primary bile acid biosynthesis, phenylalanine metabolism, metabolic pathways, cholesterol metabolism, and bile secretion metabolic pathways.

In this study, combined with the results of intestinal flora, we found that after the intervention of Jiawei Xiaoyao San, in the phylum of the JWXY group, the expression level of Firmicutes increased significantly and Bacteroidetes decreased significantly when compared with the CTL group; when compared with the PLC group, the level of Proteobacteria in the JWXY group decreased significantly. At the family level of intestinal microorganisms in each group, after the intervention of Jiawei Xiaoyao San, when compared with those in the other two groups, the contents of Porphyromonadaceae and Bacteroidales unclassified in the JWXY group decreased significantly. Compared with the PLC group, Bacteroidaceae decreased significantly. At the genus level of intestinal microorganisms in each group, when compared with those in the PLC group, Porphyromonadaceae unclassified, *Bacteroides*, and Oscillibacter in the JWXY group decreased significantly. Compared with those in the CTL group, in the JWXY group, Lachnospiraceae unclassified significantly increased and Porphyromonadaceae unclassified, Bacteroidales Unclassified, *Bacteroides*, and Oscillibacter decreased significantly.

The liver controls conveyance and dispersion, by regulating the Qi mechanism of the whole body, so that the catharsis is normal, the Qi mechanism of the body can be reached, and the Yin and Yang are balanced without the disease. In case of emotional depression, fatigue internal injury, or improper diet, it is easy to cause deficiency of Zang-Fu organs and disharmony of Qi and blood. If it is invaded by external pathogens, it can lead to qi stagnation, blood stasis, phlegm coagulation, mix deficiency, and excess, and also lead to the accumulation of symptoms over time, resulting in liver cancer. As a result of emotional stagnation, coupled with Zang-Fu deficiency damage, qi stagnation, and blood stasis, the symptoms of liver cancer are also mostly accumulation and depression, which is also an important reason why liver cancer is accompanied by emotional changes, easy to be complicated with depression, and liver depression and spleen deficiency syndrome. Bile acid is the final metabolic pathway of cholesterol, which is synthesized by liver cells. It can be divided into primary and secondary bile acids. As a messenger, it has the function of transmitting signals and can be used as the material basis for the treatment of dispersing stagnated liver qi for relieving qi stagnation. The metabolomics results indicated that the plasma levels of chenodeoxycholic acid and taurochenodeoxycholic acid in liver cancer model rats were significantly increased when compared with the control group after the successful construction of the liver cancer model rats. The level of taurochenodeoxycholic acid in the JWXY group treated with Jiawei Xiaoyao San was significantly increased when compared with that in the PLC group. Glycoursodeoxycholic acid, glycochenodeoxycholic acid, chenodeoxycholic acid, and other differential metabolites decreased significantly. After CTL and JWXY groups were compared with the model group, it was found that taurochenodeoxycholic acid was present in all their differential metabolites. Taurochenodeoxycholic acid, one of the main active substances of animal bile acids, has obvious anti-inflammatory and immunomodulatory effects and can induce apoptosis by activating the caspase cascade reaction of macrophages. Both groups showed that the increase of taurochenodeoxycholic acid may be due to tumor activation of the body’s protective effect to promote the production and release of taurochenodeoxycholic acid; therefore, the expression of taurochenodeoxycholic acid was also upregulated in the PLC group, and the expression of taurochenodeoxycholic acid was upregulated in the Jiawei Xiaoyao San intervention group, with statistical significance (*p* < 0.05). At the same time, it is noteworthy that the expression of chenodeoxycholic acid was upregulated in the model group; while in the JWXY group, the intervention of Jiawei Xiaoyao San reversed the expression of chenodeoxycholic acid. The differential metabolic pathways in the JWXY group were involved in primary bile acid biosynthesis and bile secretion. Combined with fecal microbial results, the intervention of Jiawei Xiaoyao San could significantly reduce *Bacteroides* when compared with the liver cancer group (*p* < 0.05), and *Bacteroides* are one of the participants of the main bacterial species in the intestinal microbiota of bile acid metabolism (Jia et al., 2018). The intervention effect of Jiawei Xiaoyao San on liver cancer rats with liver depression and spleen deficiency may be through regulating chenodeoxycholic acid and taurochenodeoxycholic acid, through the biosynthesis of primary bile acids and bile secretion. Regulating bile acid metabolism is important in order to play a part in liver soothing and, as a result, to fulfill the goal of liver cancer intervention to some extent.

At present, some scholars have found through the integration of clinical data that the syndrome of liver depression and spleen deficiency is one of the most common witness types of liver cancer, and it often appears in the early stage. In recent years, many doctors have conducted a large number of studies on depression based on the theory of traditional Chinese medicine and put forward that the main pathogenesis of depression is liver depression and spleen deficiency, accounting for 40.4% of all depression cases. Therefore, the study on the treatment of liver and spleen in traditional Chinese medicine is not only of great significance for the treatment of liver cancer but also can base on the physiological characteristics of the liver and spleen and the law of five elements, maintain the balance of Yin and Yang and five elements, and play an overall regulatory role, to a certain extent to prevent depression progression. In our metabolomics results, glutathione oxidized, a metabolic differential, was downregulated after the intervention of Jiawei Xiaoyao San when compared with the PLC group. Glutathione oxidized form (GSSG) and glutathione reduced form (GSH) (Arauz et al., 2016) are often used as markers of REDOX in oxidative stress. The imbalance of oxidative stress can lead to the damage of neurons and the decrease of synaptic plasticity, which can lead to depression, and has the induced risk of promoting hepatocyte gene mutation to form liver cancer (Li et al., 2015). The GSSG value (Lee et al., 2007) is highly expressed in patients with hepatocellular carcinoma and their tumor tissues, and there has been evidence that the increase of intracellular GSSG level can directly guide the death of nerve cells (Filomeni et al., 2003; Park et al., 2009). After treatment with Jiawei Xiaoyao San, the levels of Bacteroidetes, Bacteroidaceae, and *Bacteroides* in the JWXY group were significantly decreased (*p* < 0.05), and the levels of Bacteroidetes in depressed patients were significantly increased (Yu et al., 2017). Its abundance is associated with central nervous system diseases and can improve the repetitive and rigid behaviors of autistic mice by improving the microflora structure and intestinal permeability (Hsiao et al., 2013). After treatment with Jiawei Xiaoyao San, acetyl tryptophan and 5-hydroxyindole-3-acetic acid related to 5-HT metabolism in plasma was significantly upregulated. Various metabolites in the phenylalanine metabolism pathway related to dopamine metabolism also changed significantly, suggesting that Jiawei Xiaoyao San may play a role in depression relief and liver cancer treatment by regulating neurotransmitters or the central nervous system.

After treatment, the plasma content of 3-(3-hydroxyphenyl)propionate acid was significantly increased in the JWXY group; 3-(3-hydroxyphenyl)propionate acid is a flavonoid metabolite formed by human microflora. It has vasodilator activity and can play a role in reducing arterial blood pressure (Najmanová et al., 2020). Combined with the changes in fecal microbes, Oscillibacter was significantly reduced after the intervention of Jiawei Xiaoyao San. Oscillibacter is one of the harmful bacteria and is closely related to TMAO, one of the risk factors for cardiovascular disease (Huang et al., 2021), while TMAO can cause concentration-dependent arterial contraction. These results provide a basis for the role of Jiawei Xiaoyao San in promoting blood circulation in the treatment of liver cancer.

Unconjugated bilirubin (also known as indirect bilirubin) is combined with D-gluconic acid to produce conjugated bilirubin (also known as direct bilirubin). It has high solubility and low toxicity. It is mainly excreted from the body through the bile duct. Second, it can be absorbed into the blood through the small intestine and excreted from the body through renal urination. D-Glucuronic acid, produced by the decomposition of carbohydrates, can be combined with a variety of harmful substances in the liver to play a detoxification role. Compared with the JWXY group, the treated JWXY group showed downregulated D-gluconic acid. The detoxification effect of Jiawei Xiaoyao San in the treatment of liver cancer may be related to the metabolism of D-gluconic acid.

In recent years, studies have shown that the intestinal metabolites play an important role in the process of liver cancer treatment, and normal flora in the human intestine can form a protective barrier against pathogens, and intestinal flora imbalance will lead to liver cancer occurrence and development in many ways, and improving the intestinal flora can play the role of changing and even reversing liver cancer progression. In this study, we found that the analysis results of intestinal flora composition suggested that Firmicutes were the dominant phylum of the three groups, accounting for more than 50% of the total phylum, followed by Bacteroidetes and Proteobacteria. Compared with the CTL group, the composition of Firmicutes in total intestinal flora was significantly increased (*p* = 0.006), and the composition of Bacteroidetes was significantly decreased (*p* = 0.016). Compared with the PLC group, the composition of Proteobacteria was significantly reduced (*p* = 0.035). Firmicutes and Bacteroidetes, as beneficial bacteria, are the main phyla of normal human intestinal microbiota (Ley et al., 2006; Jandhyala et al., 2015). Some studies (Chen et al., 2021) show that Firmicutes and Proteobacteria are systematically reduced in intestinal microbes of liver cancer patients, while Bacteroidetes have special sphingolipids, and the imbalance of sphingolipids structure in metabolism can lead to the disorder of cell cycle (Sun and Z, 2020). The Jiawei Xiaoyao San may play a therapeutic role in liver cancer by regulating Firmicutes, Bacteroidetes, and Proteobacteria in the intestinal flora.

There is mounting evidence that Chinese herbal medicines are linked to the intestinal microbiome composition, which is linked to the conversion of Chinese herbal ingredients into active metabolites, which may have a significant impact on the therapeutic activity of Chinese herbal medicines. Microbiome analysis in conjunction with modern multi-omics platforms can identify new functional metabolites and serve as the foundation for future TCM research. For thousands of years, traditional Chinese medicine has been widely used to treat diseases in Asia. However, the lack of information on TCM mechanisms limits its application due to a lack of formal scientific verification. Traditional Chinese medicine components are frequently not absorbed directly by the host after oral administration but rather enter the intestinal tract and are transformed by intestinal flora. Intestinal flora is a community of microorganisms that live in the intestinal tract of animals and help the host in maintaining homeostasis and health.

A PubMed search revealed that there are currently only four studies that use gut microbiota and metabolomics to comprehensively analyze liver cancer, with no relevant studies on liver cancer caused by liver depression and spleen deficiency. In other comprehensive analyses, the similarities and differences between certain diseases and healthy controls were also elaborated from gut microbiota and metabolites, respectively. However, in this study, we introduced the specific syndrome types of traditional Chinese medicine and framed our research scope as the syndrome of liver depression and spleen deficiency, which increased the clarity of the research object to some extent. Through the comprehensive application of gut microbiota and metabolomics, this study provides an experimental basis for the effect of traditional Chinese medicine in the treatment of liver cancer with liver depression and spleen deficiency.

However, this research is based on the traditional Chinese medicine theory because traditional Chinese medicine normally treats ailments through a holistic and systematic diagnosis and therapy, with clinical medicines mostly in the form of a compound. Because of the complex relationship between traditional Chinese medicine compound composition, being more collaborative and antagonistic, compound system, and the disease multiple interactions between the system function and making the compound effect with complex nonlinear characteristics, it is difficult to go through specific compound mechanism or a target system individually and to clarify the specific role of traditional Chinese medicine compound mechanism. Therefore, this work did not further study and verify the specific action mechanism of Jiawei Xiaoyao San in the intervention of liver depression and spleen deficiency rats and only found the limitations of the potential action mechanism. In the future, we will consider the blood components of Jiawei Xiaoyao San and the potential therapeutic targets obtained in this study for further research, focusing on the specific functional components, target mechanism, substances, and pharmacological basis of Jiawei Xiaoyao San, to find the specific mechanism of action of TCM in the treatment of liver cancer.

## Conclusion

In this study, through comprehensive analysis of plasma metabolomics and fecal microbes on the intervention effect of Jiawei Xiaoyao San on liver stagnation and spleen deficiency liver cancer rats, some findings were proposed to reveal the potential mechanism of Jiawei Xiaoyao San. These potential mechanisms include changes in plasma metabolites, such as upregulation of taurochenodeoxycholic acid. Glycoursodeoxycholic acid, glycochenodeoxycholic acid, chenodeoxycholic acid, and other differential metabolites were downregulated, and the composition and changes of fecal microbes were adjusted, such as downregulated *Bacteroides*.

## Data Availability

The original contributions presented in the study are included in the article/Supplementary Material; further inquiries can be directed to the corresponding authors.
